# The Hypodermic Use of Pilocarpine in Serous Effusions

**Published:** 1889-04

**Authors:** L. B. Hayman

**Affiliations:** Chicago, Ill.


### THE HYPODERMIC USE OF PILOCARPINE IN SEROUS EFFUSIONS.

By L. B. HAYMAN, M. D., Chicago, Ill.*

Of the many remedies which have place in our Pharmacopeia, the greater number give results which are at best slow and obscure, while a very few have action which is marked, rapid, certain and peculiar. Among these few, the diaphoretic jaborandi stands prominent in vigor and certainty of action, for by inducing such excessive action of the salivary and sweat glands, it seems to fairly overturn the order of nature's physiology.

The drug is a comparatively new one. It was introduced from South America by Dr. Coutinho in 1874, and its alkaloid, pilocarpine, was discovered in the following year. Given by mouth or hypodermic injection, its physiological action rapidly follows. In from one to five minutes the face and neck becomes flushed and perspiration begins to ooze from the sweat glands; the droplets run together and form rivulets which course down to fall from nose, chin and ears, and the patient is busy keeping the eyes clear. The entire body is bathed in its own abundant secretion, and several pounds of perspiration may be exuded. The salivary glands pour a profuse secretion into the mouth, causing constant expectoration. The amount of saliva is at times enormous. Ringer has reported two cases in which the quantity was in one twenty-two, and in the other twenty-seven, ounces. There is free secretion from the nasal and bronchial mucous membranes and occasionally a watery diarrhœa. In nursing women there is

*Read at the March meeting of the Chicago Pathological Society.

marked increase in the amount of milk. The quantity of urine is not especially affected.

According to the authorities, the pulse rises about twenty beats; but in the case in which I have had the most experience with the drug, the heart was invariably rendered slower. There is a fall in the body temperature, as might be expected to follow such free diaphoresis. The action of the drug lasts from two to four hours, and is followed by languor and drowsiness.

There is a remarkable antagonism between the physiological actions of belladonna and jaborandi; the profuse perspiration and salivation of jaborandi being quickly checked by a hypodermic injection of atropia.

A remedy possessing such marked power over the human economy should have valuable uses in the staying of disease. Its principal field is in the induction of diaphoresis and the relief of serous effusions; and in certain cases it has marked benefit because of the vigor and certainty of its action. But the very vigor of its action has excited distrust and fear; for, it is argued, so violent a perversion of natural processes as is caused by jaborandi must result in harm, especially if long continued.

In the few years in which jaborandi has been in use by the profession it has been used in many conditions in which free salivation or diaphoresis is desirable. The reports as to its value have been various. It was at first frequently used in the renal complications of pregnancy, with the result in some cases of benefit; but in many instances its use was followed by pulmonary congestion and a fatal result. Because of these reports the drug has perhaps fallen into greater disuse than its physical effects merit.

Organic disease of the heart is the main contra-indication to the use of jaborandi, because of its marked depression. The use of the infusion or fluid extract frequently causes nausea, and the most satisfactory results follow the hypodermic use of the muriate or nitrate of pilocarpine, in doses of from ¼ to 1 grain.

Bartholow speaks of it as of great value in cardiac dropsy, if there is no affection of the gastro-intestinal mucous membrane, or disease of the muscular substance or ganglia or valves of the heart; of the quick removal by its use, of effusions of pleuritis, hydro-thorax and ascites; of its value in uræmia, and in renal dropsy, especially scarlatinal, if not contra-indicated by some disease of the heart.

In Ringer's Therapeutics, jaborandi is spoken of as useful, especially in the uræmic symptoms of Bright's disease. It is not a remedy of transient value only, for he records having seen marked improvement follow hypodermics of pilocarpine.

G. Baumgarten, in the "Reference Handbook of the Medical Sciences," speaking of the complication of complete anuria in the course of acute parnechymatous nephritis, advises the use of hypodermics of pilocarpine, grs. 1-12 to ⅓, according to age. But adds that it is contra-indicated in cases of weak heart and of respiratory complications, and must be used with caution. In chronic parenchymatous nephritis, he refers to pilocarpine as an exceedingly valuable remedy. The renal affections of pregnancy, according to this author, are not to be treated with pilocarpine. Speaking of contracted kidney, he says: "When uræmic stupor or coma sets in, the hypodermic use of pilocarpine is perhaps the only means of staying the rapid progress of the case toward the fatal end."

I. T. Dana, speaking of dropsy, writes: "Jaborandi and pilocarpine stands at the head of diuretics. They may be used daily for a succession of weeks if needed. The nitrate of pilocarpine may be given hypodermically in doses of gr. ⅛ to ¼, the patient being in bed between blankets, a very profuse and general sweating is usually the prompt result, with free elimination of uræ."

In his recent lectures on "Albuminuria," T. Grainger Stewart, of Edinburg, advises in acute uraemia the use of pilocarpine hypodermically, in conjunction with hot air baths.

In Loomis' "Practice," jaborandi is spoken of as having been much employed for the removal of dropsical accumulations; but the author goes on to say: "In most cases these accumulations can be rapidly removed by this drug, but my own experience leads me to the conclusion that it hastens rather than retards the fatal issue." In the urgent symptoms of chronic parenchymat_ ous nephritis, also, he advises caution in the use of jaborandi or pilocarpine, characterizing them as prompt and efficacious, but sometimes dangerous.

In his work on Renal Diseases, William H. Porter speaks of the value of jaborandi in the treatment of chronic parenchymatous metamorphosis as follows: "In regard to this medicinal agent, much has been said *pro et con*, and in lesions of this type it has been used with varying doses, some claiming that it has a depressing, others that it has a stimulating, influence upon the heart; but the truth is that in small quantities it appears to depress and in large doses to stimulate that organ. From observation in many of the cases upon which this chapter is based, only one conclusion can be deduced, namely, that in most instances the quantity administered is too small. The dose as commonly given in most of the works on therapeutics is from 1-5 to ½ grain of pilocarpine. In all cases from which these conclusions are formulated, the drug was given by subcutaneous injection, and the smallest amount ever administered was ½ grain of muriate of pilocarpine. This preparation is preferable to the nitrate or sulphate, and with this dose the effects were not very marked; but when the quantity was raised to ⅔ to ¾ grain, the most desirable results were obtained."

He cites a case of a patient in convulsions in whom the dropsy was so marked that he was fairly water-logged. Two grains of the muriate of pilocarpine were given hypodermically, followed soon by another. The convulsions and coma wore away, the œdema partially disappeared, and consciousness returned, so that for eleven days the patient was fairly comfortable. He died on the twelfth day from exhaustion, with no return of the convulsions.

Dr. Porter adds: "Continued observation has failed to reveal any case where there was evidence of a depressing effect from the use of this drug, but owing to the fact that the opposite has been reported, it is always well to give some ammonia or alcohol, or both, in advance of the drug."

I have used pilocarpine in a number of instances, both as a galactagogue and as a diaphoretie, and in one case the use of the drug was continued for several months with most gratifying results.

*Case.*—The patient was a young lady of seventeen, who came to the city from Earlville, Illinois, suffering from chronic Bright's disease. She was under the care of Professor I. N. Danforth, who upon leaving the city for a time, placed her in my charge, and I saw her for the first time on the 5th of September, 1887. She had been sick for about eight months, and was in the later stages of chronic nephritis. There was extreme œdema of all the cellular tissues of the body, so that the patient could not move from her chair. Both pleural sacs contained fluid, the respiration was difficult and there was a harsh dry cough; the heart was labored in its action. For days the patient had been unable to lie down, and spent the nights in her chair, catching an occasional short nap with her head resting upon a pillow on her lap. She was restless and nervous, although very patient. But little nourishment could be retained, and the end was apparently so near that relatives had been summoned.

Diaphoresis had been invited by hot air baths, and diuretics and hydragogue cathartics had been freely used; but still the dropsy increased. The swollen feet had been tapped twice, allowing of considerable escape of serum, with but temporary benefit.

An examination of the scanty urine showed an immense deposit of albumen, about three-fourths of the contents of the test tube solidifying with heat. The urine was of high specific gravity, and showed, under the microscope, a large number of blood corpuscles, clouds of granular and epithelial casts, with renal epithelium.

The weak, rapid heart, and the labored respiration both contraindicated the use of pilocarpine, but other remedies had failed and it offered the only chance of benefit, so I decided to use it. The patient was placed in bed—seated, for she could not lie—and a hot-air apparatus was placed under the blankets. She was given half an ounce of brandy, and then a hypodermic injection of $\frac{1}{3}$ of a grain of the muriate of pilocarpine. In about a minute the constitutional effects of the drug appeared, and soon there was an abundant secretion of saliva and the attendants were busy wiping the streams of perspiration from her face and neck. There was coughing with free expectoration of mucus; the respirations became easier, and the pulse, which had been about 120 to the minute, fell to 100.

The patient expressed herself as wonderfully relieved. The perspiration lasted about two hours and was followed by a quiet night. The injection was repeated on the following day with similar results. In each case the sweating was profuse and was followed by a marked diminution in the nervous symptoms. The injections were given daily for several weeks, accompanied by iron, digitalis and hydragogue cathartics. Under this treatment the dropsy steadily diminished, so that soon the patient could lie down; the nervousness was lessened, and there was a gain in appetite and strength. The urine was also improved. The amount of albumen was much diminished and the casts, at first so numerous, were found with difficulty. There seemed to be real improvement, not only in the general symptoms, but in the condition of the kidney as well.

The gain was so gratifying that, after about four weeks, the patient, feeling sure of ultimate recovery, began to speak of returning home. She thought the treatment might still be carried on at her home in Earlville, and on the 5th of October, contrary to advice, she took the train for home.

Because of the condition of the heart, her home physician, Dr. E. T. Goble, of Earlville, Illinois, would not use the pilocarpine until after he had communicated with me, and in the two or more weeks which intervened, the dropsy rapidly returned. The hypodermics were then recommended, and persisted in almost daily until her death, which occurred on the 26th day of May following, about eight months later.

The pilocarpine seemed always to benefit, and the patient would plead for the hypodermics. They always relieved the nervousness, probably by the elimination of urea, quieted and slowed the heart and gave ease and comfort. It is quite certain in this case, that while it did not avert the fatal issue, the use of the drug gave comfort, and prolonged life for many months.

